# The role of the pulmonary function laboratory in the management of gastrointestinal and liver diseases

**DOI:** 10.36416/1806-3756/e20240350

**Published:** 2024-12-17

**Authors:** José Alberto Neder, Denis E O’Donnell, Danilo C Berton

**Affiliations:** 1. Pulmonary Function Laboratory and Respiratory Investigation Unit, Division of Respirology, Kingston Health Science Center & Queen’s University, Kingston (ON) Canada.; 2. Unidade de Fisiologia Pulmonar, Hospital de Clínicas de Porto Alegre, Universidade Federal do Rio Grande do Sul, Porto Alegre (RS) Brasil.

## BACKGROUND

The respiratory and gastrointestinal (GI) systems share several embryogenic, anatomical, and physiological features. Pulmonary function tests (PFTs) are beneficial in detecting respiratory consequences of advanced liver disease and inflammatory bowel diseases (IBDs, mainly Crohn’s disease and ulcerative colitis), which have potential implications for management.^1^

## OVERVIEW

A 58-year-old man with cirrhosis secondary to long-term alcohol abuse (patient A) was referred for PFTs because of worsening dyspnea despite apparently stable end-stage liver disease. Mild intraparenchymal restriction was associated with severe impairment in DL_CO_ (adjusted for hemoglobin) and carbon monoxide transfer coefficient. A markedly enlarged alveolar-arterial oxygen gradient (44 mmHg) coexisted with moderate to severe hypoxemia (PaO_2_ = 51 mmHg) and hypocapnia, plus mixed respiratory and metabolic alkalosis. Dyspnea and hypoxemia worsened from supine to seated position (i.e., the patient presented with platypnea-orthodeoxia syndrome). Testing with 100% inhaled oxygen revealed a shunt fraction of approximately 20%; extensive intrapulmonary vascular dilatations were confirmed with contrast-enhanced echocardiography and computed tomography (CT). A 49-year-old woman with Crohn’s disease (patient B) reported three episodes of acute-on-chronic dyspnea and productive cough, together with worsening GI symptoms, over a one-year period. Sequential testing showed recurrent relapses of mild-to-moderate mixed obstructive and restrictive defects with air trapping seen on PFTs (high RV and high RV/TLC ratio) and on inspiratory-expiratory chest CT.

**Figure 1:**
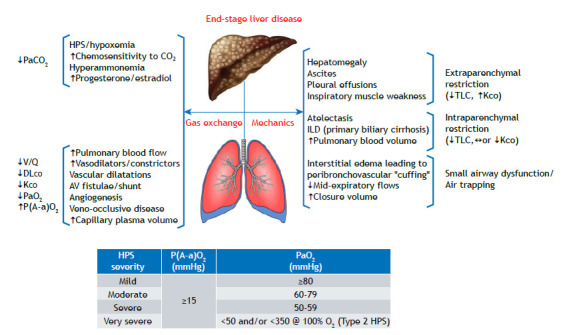
A simplified overview of the main respiratory consequences of end-stage liver disease and their impact on common pulmonary function test results. The table depicts the gas exchange criteria used in grading the severity of hepatopulmonary syndrome. V/Q: (alveolar) ventilation/perfusion ratio; K_CO_: carbon monoxide transfer coefficient; P(A-a)O_2_: alveolar-arterial oxygen gradient; AV: arteriovenous; ILD: interstitial lung disease; ¯: decreased; ­: increased; and «: preserved.

Hypoxemia in liver disease is strongly related to abnormal function of the pulmonary (micro)vasculature, secondary to either the production of or failure to clear a broad range of inflammatory, vasoactive, or proliferative/angiogenic mediators (Figure 1).^2^ Hepatopulmonary syndrome, as was seen in patient A, is a gas exchange disorder characterized by severe arterial hypoxemia, occurring in up to a quarter of patients with cirrhosis or portal hypertension. The syndrome is caused by intrapulmonary vascular dilatation and can be subdivided into two types: type 1, causing diffusion-perfusion defect; and type 2, causing anatomic shunt. When the vasoconstrictive/proliferative consequences dominate, a smaller fraction (2-8%) of patients with cirrhosis develop portopulmonary hypertension. Characteristically, hypoxemia is less severe unless advanced pulmonary hypertension leads to the right-to-left shunt through a patent foramen ovale.^3^ Because of the multiple mechanisms related to a low DL_CO_ (Figure 1), the expected pattern of extraparenchymal restriction (i.e., low TLC and supra-maximal carbon monoxide transfer coefficient) may not emerge in some patients despite voluminous ascites.

The spectrum of lung involvement in IBDs is broad, ranging from subclinical abnormal involvement to interstitial lung disease, airway disease (panbronchiolitis, bronchiolitis obliterans organizing pneumonia, and bronchiectasis), as was seen in patient B, inflammatory tracheal stenosis, vasculitis, pleural disease, and enteric-pulmonary fistulas, among other conditions. As such, the PFT abnormalities are heterogeneous, reported in 17-55% of patients.^4^ Lung abnormalities can be present years following the onset of the disease, commonly occurring during active disease and even post-colectomy. The underlying pathogenesis may be associated with the fact that the colonic and respiratory epithelial cells share a common embryonic origin and similar mucosal immunity.

## CLINICAL MESSAGE

Respiratory function should be assessed in all candidates for liver transplantation, given that primary disorders affecting the pulmonary circulation (hepatopulmonary syndrome and portopulmonary hypertension) are associated with poorer functional status, worst survival while on the waiting list, and unfavorable outcomes after the procedure.^5^ Abnormal PFT findings may signal acute deterioration of IBDs even in the absence of overt respiratory symptoms: concomitant improvement in GI symptoms and lung function is not uncommon, illustrating the complexities involved in the lung-gut axis.^1^
